# The History of Left Septal Fascicular Block:  Chronological Considerations of a Reality Yet to be Universally Accepted

**Published:** 2008-04-01

**Authors:** Andres Ricardo Perez Riera, Augusto Hiroshi Uchida, Edgardo Schapachnik, Sergio Dubner, Li Zhang, Celso Ferreira Filho, Celso Ferreira, Dardo E Ferrara, Antoni Bayes de Luna, Paulo Jorge Moffa

**Affiliations:** 1 Electro-Vectorcardigraphic Section, ABC Medical School, ABC Foundation, Santo Andre - Sao Paulo, Brazil; 2 Electrocardiology Service, Heart Institute (InCor) of the University of Sao Paulo Medical School, Sao Paulo, Brazil; 3 Department of Chagas Disease, Dr. Cosme Argerich Hospital, Buenos Aires, Argentina; 4 Arrhythmias and Electrophysiology Service, Clinical y Maternidad Suizo Argentina, Buenos Aires, Argentina; 5 LDS Hospital University of Utah School of Medicine, Salt Lake City UT, USA; 6 Cardiology Division, ABC School of Medicine, ABC Foundation and School of Medicine of Santo Amaro - UNISA - Sao Paulo, Brazil; 7 Cardiology Division, ABC School of Medicine, ABC Foundation - Santo Andre - Sao Paulo - Brazil; Professor of the Federal University of Sao Paulo - Sao Paulo - Brazil; 8 Cardiology Division, Emory University School of Medicine, Atlanta, GA, USA; 9 Autonomous University of Barcelona, Institut Catala Ciencies Cardiovasculars, Hospital Sant Pau, Barcelona, Spain; 10 Electrocardiology Service, Heart Institute (InCor) of the University of Sao Paulo Medical School, Sao Paulo, Brazil

**Keywords:** Left Hissian intraventricular system, Fascicular blocks, Left Septal Fascicular Block, Hemiblocks

## Abstract

There are several papers in literature that prove in a conclusive and incontestable way, that the left branch of the His bundle, in most instances (85% of the cases) splits into three fascicles of variable morphological pattern, and not into two: left anterior fascicle (LAF), left posterior fascicle (LPF), and left septal fascicle (LSF). The abovementioned papers have anatomical, histological, anatomo-pathological, electrocardiographic, and vectocardiographic, body surface potential mapping or ECG potential mapping and electrophysiological foundation.

Additionally, the mentioned papers have been performed both in animal models (dogs) and in the human heart.

Several clinical papers have shown that the left septal fascicular block (LSFB) may occur intermittently or transitorily as a consequence of a temporary dromotropic alteration, constituting an aberrant ventricular conduction, rate-dependent or by the application of atrial extra-stimuli, or naturally during the acute phase of infarction when this involves the anterior descending artery, before the septal perforating artery that supplies the central portion of the septum, where the mentioned LSF runs.

The ECG/VCG manifestation of LSFB consists in anterior shift of electromotive forces, known as Prominent Anterior Forces (PAF), which can hardly be diagnosed in the clinical absence of other causes capable of causing PAF, such as the normal variant by counterclockwise rotation of the heart on its longitudinal axis, in right ventricular enlargement, in the dorsal or lateral infarction of the new nomenclature, in type-A WPW, in CRBBB, and others. In this historical manuscript, we review in a sequential fashion, the main findings that confirmed the unequivocal existence of this unjustifiably "forgotten" dromotropic disorder.

In the developed countries, its most important cause is coronary insufficiency, particularly the proximal involvement of the left anterior descending coronary artery, and in Latin America, Chagas disease.

## The trifascicular background of the left Hissian Intraventricular system

The story begins at the end of the 19^th^ century with the initial description by Dr. Whilhelm His Jr. of the left Hissian intraventricular system as being trifascicular [1]. He showed that a connective tissue sheet became a bundle connecting the upper and lower cardiac chambers, the bundle of His [2].

**Figure 1:**
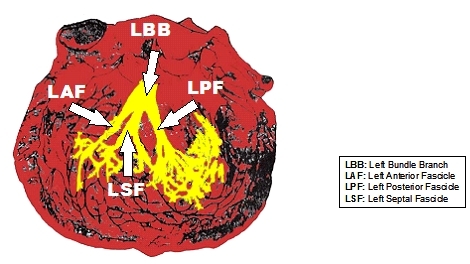
The Trifascicular Nature of the Left His System. The trunk of the left bundle branch (LBB) of the His bundle split in three fascicles: Left anterior fascicle (LAF), Left septal Fascicle (LSF) and Left Posterior Fascicle (LPF).  "The Conduction System of the Mammalian Heart" (1906)

At the beginning of the 20th century, Dr. Suano Tawara clearly showed that the trunk of the left bundle branch (LBB) split in three fascicles and not in two [3,4] (Figure 1).  Tawara's pioneering work on the human conduction system still serves as an invaluable reference for basic and clinical research [5].

The electrocardiographic study of divisional blocks of the left His system goes back to 1917 with the work by the German researchers Rothberger and Winterberg [6]. These authors observed that the injury in the divisions of the LBB produced significant deviations of SÂQRS in the frontal plane (FP), besides a discrete increase in QRS duration.

On the basis of experiments in dogs in 1956, Scher and Young [7] found that the initial activation of the septum occurred in three different areas.

In 1960, Uhley et al [8,9] described the trifascicular characteristic of the canine left His system and four years later, they showed the electrocardiographic manifestations (also in dogs) of the interruption of the trunk of the left branch and its three divisions. In the same year, Robb [10] reinforced the trifascicular characteristic of the left His system with his anatomical studies.

In 1967, Mauricio Rosenbaum [11-15] and his school made a great contribution to the study of this matter and had a great influence on the world of arrhythmology. He brilliantly described the electrocardiographic manifestations of blocks in the antero-superior (LAFB) and postero-inferior (LPFB) divisions of the LBB. This school considered that the human left His system was anatomically and functionally bifascicular, and for this reason the term hemiblocks was coined: left anterior hemiblock corresponding to the current LAFB and left posterior hemiblock corresponding to LPFB. These authors maintained the same nomenclature until to day. One year after the first Rosenbaum's publication, and contrary to his school of thoughts, Barry and Pattern [16] showed the trifascicular structure of the left His system. In that same year, in an electrocardiographic and vectorcardiographic study Cohen et al [17] demonstrated the variations of aberrant ventricular conduction in man with evidence of isolated and combined block within the specialized conduction system.

**Figure 2:**
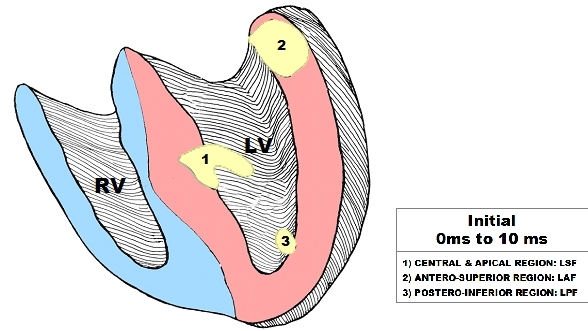
Sequence of 10ms Initial Normal Ventricular Activation

In 1970, Dr. Dirk Durrer et al [18] from the Departments of Cardiology and Physiology, Amsterdam, established in a classical manuscript, using 870 intramural terminals in the isolated human heart that three endocardial areas were synchronously excited from 0 to 5 ms after the start of the left ventricle (LV) activity potential. To obtain information concerning the time course and instantaneous distribution of the normal excitatory process, the authors studied isolated hearts from seven individuals who died from cerebral conditions but had no history of cardiac disease (Figure 2).

The LV areas first excited were:

1. High on the anterior paraseptal wall just below the attachment of the anterolateral papillary muscle (ALPM) where end the LAF;
2. Central on the left surface of the intraventricular septum (IVS) where end the LSF. Septal activation started in the middle third of the left side of the IVS, somewhat anteriorly, and at the lower third at the junction of the IVS and posterior wall. The normally functioning LSF, the left middle septum surface and the inferior two-thirds of the septum originate the first vector, vector 1 or first anteromedial (1AM) vector and left inferior two-thirds of the IVS (second vector or vector the inferior two-thirds of IVS) [19];
3. Posterior paraseptal, about one third of the distance from apex to base near the base of PMPM where end the LPF. The posterobasal area is the last part of the LV to be activated. Finally, endocardial activation of the right ventricle (RV) was found to start near the insertion of the anterior papillary muscle of the tricuspid valve 5 to 10ms after onset of the LV cavity potential. This initial activation of RV apex is coincident with the activation of inferior two-thirds of the left septum. Because the last one has opposite direction and has a bigger mass the RV apex activation is not manifested.

One year later in Italy (1971), Rossi [20] reinforces the trifascicular nature of the left intraventricular His system with a new anatomical and histopathological study.

Demoulin and Kulbertus et al [21,22] performed a histopathological viewpoint study of the LBB system in hearts from patients without conduction defects and found that the LBB would give off a third radiation or branch in 11 out of the 20 hearts. This structure traveled to the midseptal area and emerged either from the common left bundle (5 cases), from the anterior (3 cases) or posterior radiation (3 cases).

In 1972-73, Uhley [23-25] emphasized the same concepts in a classical editorial and a book chapter.

In 1972, Myerburg et al [26], working on isolated canine hearts, proved the trifascicular nature of the left His system by the mode of verified endocardial excitation. The same author would stress these ideas three years later [27,28].

The terminology of hemiblocks is criticized for the first time in 1973 by Hecht et al [24] coined the terms divisional/fascicular blocks as being more appropriate, since it was clear that the left branch split in three and not in two branches.

In that same year, Gambeta [29] and Childers described the first electrocardiographic manifestations secondary to septal fascicular or septal focal block. These authors verified the intermittent, rate related appearance of Q waves in right precordial leads in the absence of antero-septal infarction. They also highlighted the frequent association with RBBB and/or LAFB.

Lazzara et al [30] in 1974 mapped canine left ventricular endocardial surface in vitro before and after lesions were placed in the proximal left bundle branch and proved the independent functional and anatomical behavior of the middle fibers of the LBB. In a subsequent publication, this author observed different types of refractoriness of the intraventricular system with three well-defined pathways. Additionally, the action potential and absolute, relative and functional refractory periods are significantly shorter in the LSF when compared with LAF and LPF. Phase 0 of the LSF is wider and consequently, conduction velocity is greater  which justifies the centro-septal region activating 5 ms before the anterior and posterior ones. The three have an automatic phase 4, i.e. with discrete spontaneous elevation or diastolic depolarization [31].

Kulbertus [32,33], in successive publications from 1975 and 1976, showed the distribution of the left branch and its three fascicles: anterior, posterior and septal. He studied 49 human heart specimens and found that only in 16 of them (15%) the third fascicle was missing, i.e. they were bifascicular. The remaining 85% was grouped in three types:

**Type I:** In 33 cases (74%), the septal division was easily identifiable, possible of a diameter greater than the other divisions, originating from the trunk of the left branch (18 cases), the antero-superior division (7 cases) or the postero-inferior division (9 cases).

1. Centro-septal area, in charge of a division of very variable and inconstant anatomy;
2. High anterior paraseptal area, just below the insertion of the septal fascicle of the mitral valve, which ends in the anterior papillary muscle.

**Type II:** In 11 cases (23%), the author observed that there was a net made up by several fascicles coming from the antero-superior and postero-inferior divisions.

**Type III:** In 5 cases (2.4%), branch of the postero-inferior division with a prolongation of "false tendons" of the postero-inferior division.

**Figure 3:**
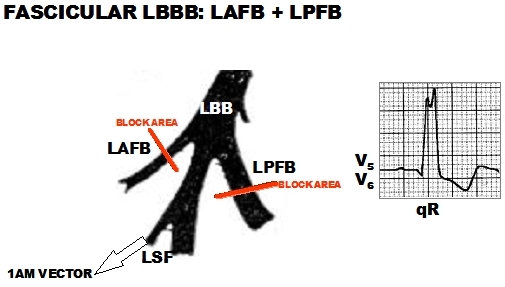
Atypical LBBB with initial q Waves in Left Precordial Leads

Then, we should ask why the initial ventricular activation (10 ms) occurs in three points of the left septal surface and not in two, as it should be expected if the left His system were functionally bifascicular? Moreover, how to explain the cases of left bundle branch block (LBBB), divisional blocks (LAFB + LPFB) that present q wave in left leads, outshining the typical electrocardiographic pattern of LBBB? Mauricio Rosenbaum called them "left intraventricular blocks without changes in the initial part of the QRS". The great master, in his colossal work, states that these cases are "hard to explain" [11]. In 1970, Medrano et al [34] proposed that in these cases, the fibers of the septal division would originate before the location or area of block in the postero-inferior or antero-superior divisions. As a result, middle-septal activation is preserved (1AM vector) and is responsible for those q waves in left leads and concealing the LBBB pattern. In other words, by totally blocking the two antero-superior and postero-inferior divisions, LBBB did not occur, as it should be expected in a situation where there are only two branches (Figure 3).

In 1976, Massing and James [35] examine histologically the conduction system of 13 human hearts. They found that the LBB anatomy was extremely variable, demonstrating multiple fiber groups which fanned out over the entire left septal surface.

In that same year, Kulbertus [ studied the phenomenon of aberrant ventricular conduction after the provocation of premature atrial extra-stimulation with transvenous catheter electrode. The most common types of aberration were as follows: isolated RBBB in 28 patients, RBBB with LAFB in 21, LAFB in 17, RBBB along with LPFB in 10, LBBB in another 10 cases, and incomplete LBBB in 6 patients. In 5 patients, an important anterior displacement of vectocardiographic QRS loop was observed in the horizontal plane, maintaining counterclockwise rotation and with more than 75% of the area of the loop in front of the X line. Three of these five cases had normal previous VCG, one had a dorsal infarction and an anterior infarct was present in the last one.^36^]

Subsequently, Hoffman et al [37] showed in a series of clinical-electro-angiographic cases, several examples of LSFB translated electro-vectocardiographically into significant displacement of the ventricular depolarization forces to the front in the horizontal plane.  Interestingly, this author explained the mechanism as a conduction delay in an anterior division of the left bundle branch system.

The Portuguese school led by Fernando De Padua [38,39] mentioned that an anterior displacement of the QRS loop, independent of the axis deviation in the FP, can be observed and may be a result of the involvement of centroseptal fibers. The ECG pattern is supposed to show prominent R waves in V1-V2 similar to those found in dorsal infarction concomitant with no abnormal axis deviation in the FP [40].

In 1977 a classical book of electro-vectocardiography gave a detailed explanation of the new concepts of septal activation and conduction delays. The initial vector (first 0.01s) represents the activation of the middle third of the left septal surface, and the vector of the 0.02 s, the apical-anterior region, which is oriented to the front and the left, and the delay in the apical area could cause anterior displacement of the QRS loop [41], meanwhile, during this same year, Alboni followed a case of aortic regurgitation that initially presented with an ECG pattern of left ventricular enlargement and repolarization abnormalities ("strain") [19]. One year later, ECG and VCG revealed divisional LBBB (LAFB + LPFB); though, maintaining septal activation (Q waves in left leads). This phenomenon was attributed to preservation of conduction by the middle septal fascicle.

In the late 70s' in Japan, Nakaya et al [42,43] demonstrated the presence of prominent anterior forces (interpreted as LSFB) in association with coronary artery disease. Later on, the same authors carried out experiments in dogs to prove this concept.

These authors have also proposed that these prominent anterior forces are frequently seen in hypertrophic cardiomyopathy (HCM), particularly in the *non-obstructive form*. These cases may also represent true LSFB. The broad R waves from V2 to V4 and QRS loop in the horizontal plane with more than 2/3 in front of the X line associated to absence of initial convexity (initial 20 ms) to the right is attributed to the involvement of the left branch divisions (LSF and LASF), caused by hypertrophy and fibrosis of the septum. Nakaya analyzed 1000 consecutive ECGs and VCGs and found three cases of HCM with prominent anterior forces, two being non-obstructive forms and one obstructive.

In 1978, Iwamura et al [44] performed experiments in isolated canine hearts and provided evidence for the different electrophysiological properties of the left septal Purkinje arborizations in the face of premature stimulation. During 1978-79, Dabrowska et al [45,46]. attempted the standardization of diagnostic ECG criteria of LSFB in anesthetized and intubated dogs after incision of the interventricular septum.

**Figure 4:**
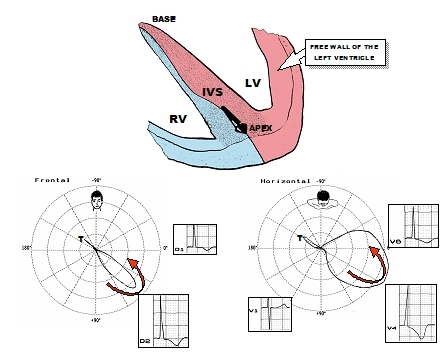
VCG of a non-obstructive form of HCM. The septum (S) and left ventricular free wall (FW) are thicker in their apical portions (FW), i.e. there is absence of normal decrease in the thickness from the base to the apex. The author suggests that the anterior and left dislocation of the QRS loop in the horizontal plane, and inferior and to the left in the FP (translated by R waves of greater voltage in V4 and DII) are secondary to selective hypertrophy of the apical inferior third of the septum. The absence of q in left leads was explained by ILBBB or LSFB.

In 1979, Cheng et al [47] observed that high QRS voltage in midprecordial leads was a frequent finding in non-obstructive forms of hypertrophic cardiomyopathy. The ECG/VCG patterns were very suggestive of Left Septal Fascicular Block with prominent anterior forces. The author attributed the absence of q waves in left leads (85% of the cases) to incomplete LBBB or LSFB (Figure 4).

Also in 1979, Athanassopoulos [48] presented a case of an acute myocardial infarction of the infero-anterior wall showing Q waves in the inferior leads and from V2 to V4, which disappeared twelve hours later. The myocardial infarction was confirmed by a typical enzymatic curve. The author attributed the ECG changes to transitory block of the septal region.

In the same year, the Brazilian school led by Prof. Joao Tranchesi [49] exemplified with great detail the electro-vectocardiographic aspects of isolated LSFB, and the more frequent cases seen in association to other intraventricular blocks, both in coronary insufficiency and Chagas disease.

In 1980, followers of the Tranchesi School [50] published a clinical-electro-vecto-angiographic correlation of the dromotropic disorder of the septal division associated with critical injury of left anterior descending artery. The authors found prominent anterior forces in V1 and V2 (even R/S >1 in V1) in patients without posterior involvement and confirmed critical injury of the left anterior descending artery. In particular, if primary T wave abnormalities were present, ischemic LSFB should be suspected.

In 1981, Nakaya and Hiraga studied this phenomenon, by producing cuts in the Purkinje arborizations of the middle septal region of dogs [43,51].They verified anterior and left dislocation of the QRS loop, along with activation delay in the apical region. Accordingly, the block in this area (11 dogs) resulted in a discrete delay in the apical area activation and in the Z lead (postero-anterior and corresponding to the V2 line), and an increase in voltage was observed from 1.4 to 2.6 mV by dislocation of the QRS loop to the front. Additionally, the block in the postero-inferior division (9 dogs) caused discrete delay in activation of the postero-basal region. On the other hand, the concomitant block in both areas (middle-septal and postero-inferior: 11 dogs) caused an important delay in the activation of an extensive area in the postero-basal and apical regions, significantly affecting the axis of X with the appearance of S and important deviation to the right of AQRS in the FP. The authors concluded that the Purkinje central septal block per se might cause significant electro-vectocardiographic changes by its capacity of interconnection. In all the cases that the left conduction system was anatomically analyzed, wide morphological variability was found. Interestingly enough, the LBB was never organized in two well-defined fascicles.

The main disciples of the Tranchesi School, Prof. Moffa et al [52,53] have showed in several publications the existence of LSFB, both in Chagas disease and in coronary artery disease. These authors have the merit of illustrating the intermittent character of the phenomenon.

In 1983, Inoue et al [54] in a vectocardiographic experimental work in dogs, verified that causing a block in the distribution area of the septal fascicle ("septal Purkinje network" called by the authors as SEP) a discrete delay is produced in epicardial activation of the septal area and in a 30% of instances an anterior dislocation of the QRS loop in the HP occurs. When the block was caused in the region of the antero-superior division, significant changes were not observed in VCG, with only superior and left deviation of the terminal vector of QRS being recorded. Finally, when the block was produced in both regions, the delay in activation reached an area much greater, from the anterior basal region up to the apical area and the direction of the maximal vector in the FP showed a significant superior and left dislocation. The authors concluded that the block in the SEP area per se, produces electrical changes with sufficient traits to be characterized.

Sakaguchi et al [55] in 1988 performed an autopsy study of the left intraventricular system in 13 normal human hearts obtained from subjects aged 50 to 80 years. The samples were stained with hematoxylin-eosin or by the van Gieson method and examined by light microscopy. Reconstruction was performed using a two-dimensional system in order to histologically differentiate the bundle cells from Purkinje cells. The LBB bifurcated into the anterior and posterior radiations and the cells in the septal portion were almost all Purkinje cells except in two cases showing a septal branch between the two radiations. This difference in structure may explain why the centro-septal region is activated 5 ms before the others.

In 1992, Mori et al [56] proposed criteria for the diagnosis of left septal fascicular block, based on the normal limits of the R and S waves and R/S of V1 and V2. The criteria can be summarize as follows:

1. Exclusion of other causes responsible of anterior QRS displacement, such as normal variant, chest anomalies (counterclockwise rotation of the heart in the longitudinal axis), type A RVH, RBBB and dorsal infarction.
2. One of the following two voltage criteria should also be satisfied. (i) R/S in V1 > 2, and R in V1 ≥ 5 mm, (ii) R/S in V2 > 2, and R in V2 ≥ 15 mm, or S in V2< 5 mm. The frequency of left septal fascicular block diagnosed by these criteria was 3.5% among a large group of hospitalized patients. This frequency was less than that of the LAFB or RBBB, but it was higher than that of bilateral BBB.

In an elegant study published in 1996, Dhala et al [57]. demonstrated the trifascicular nature of the left His system during catheter ablation of the right branch. Twenty-five patients underwent catheter ablation of the RBB, either for bundle branch reentrant tachycardia, or inadvertent or deliberate right bundle ablation during atrioventricular junctional ablation for rate control.  These authors divided all the patients (25) in two groups:

Group I: the patients in this group (n=11) did not have previous signs of pre-ablation dromotropic disorder. After the ablation of the right branch only RBBB emerged.

 Group II: made up by those patients (n=14) with some right bundle branch conduction delay that was present before the ablation of the right branch. In 12 out of 14  a qR pattern in V1 was observed and interpreted as LSFB associated to AQRS deviation, either to the right (LPFB: 3 patients) or left (LSFB: 4 patients). The authors concluded that the trifascicular nature of left intraventricular conduction became apparent, or was unmasked, after involvement of the right branch.

In 1997, Moffa [58,59] presented an undisputable case  of a 69 year old patient with severe three-vessel disease who initially presented with the following ECG: sinus rhythm, first-degree AV block, QRS duration of 110 ms, QRS axis in 0º, Q waves from V1 to V6, followed by broad R waves from V1 to V4, which decreased voltage in V5 and V6. The vectocardiogram presented prominent anterior forces of the QRS loop, with middle-area vector in the horizontal plane in +60º. This pattern was interpreted as LSFB in absence of other causes that may determine prominent anterior forces. Two years after, the patient was readmitted with acute pulmonary edema and cardiogenic shock due to a myocardial infarction. The ECG  had important changes  after two days: QRS ( 160 ms), QRS vector of +100º, R waves in leads II, III and  AVF that increase from II to III, QS in lead 1, AVL, AVR and V1, rS in V2 and V3, R wave was notched in V5 and V6. This was a transient pattern that returned to the initial one in 24 hours.

In 2001, Sanches, Moffa and Sosa [60] from the InCor, Sao Paulo, Brazil studied five patients that met ECG/VCG criteria of LSFB, who underwent biventricular endocardial catheter mapping. These ECG/VCG patterns were characterized by prominent anterior forces with increased ventricular activation time in V1 and V2, small initial q wave in V2-V3, R wave of V2 and V3 >15 mm, sharp-pointed R wave in V2-V3 leads with slow descendent ramp, absence of q wave in left precordial leads V5, V6 and I (by absence of the vector 1AM), increasing voltage of R waves in V3-V4 and decreasing from V5 to V6. The QRS loop of VCG in the HP showed a marked anteriorization of the QRS loop, which is located predominantly on the left anterior quadrant and has a CW rotation. The authors observed conduction delay in the middle left septal surface and in the free wall of the LV - named anterior conduction delay - (sites 9, 10, 11 and 12 from the original description by Josephson et al [61]. These sites correspond to the area of distribution of the LSF.

Recently (2002-03), Prof. Rex MacAlpin [62,63] from Division of Cardiology at UCLA, presented possible models of LSFB ventricular activations, proposed diagnosis criteria and showed illustrative cases of probable and possible LSFB based on deductive reasoning. The author concludes that the LSFB is a polymorphic conduction defect which may explain some previously inadequately understood electrocardiographic abnormalities.

**Figure 5:**
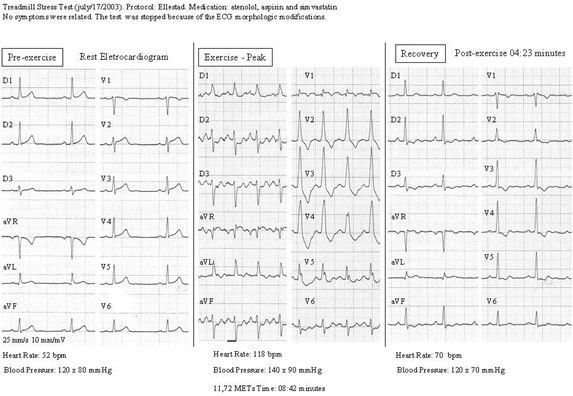
Exercise-induced left septal fascicular block: an expression of critical obstruction of left anterior descendent artery. Tall R waves in V1-V4 can be a normal variant in only 1% of patients and it is a hallmark ECG finding in left septal fascicular block. The proposed ECG criteria for LSFB are: prominent R waves in V1-V3 (Prominent anterior forces or PAF), minimal QRS prolongation (QRS < 120 ms), T wave morphologic alteration (flat or inversion: very debatable and variable), frequent initial q wave in right and/or middle precordial leads and clinical absence of other causes of PAF [64].

Finally, we have recently presented a case report of intermittent exercise-induced left septal fascicular block as an expression of severe myocardial ischemia, with critical obstruction of left anterior descending artery [64] (Figure 5).

## Conclusions

The trifascicular nature of the human left His system has been a controversial matter. After conducting an extensive chronological review on this subject, we conclude that in most cases, the left His system is trifascicular. In fact, the LSFB has distinct ECG and VCG patterns. It is characterized by prominent anterior forces on the horizontal plane, which can be distinguished from the normal variant with counterclockwise rotation of the heart around the longitudinal axis, types A or B patterns of RV enlargement,  misplaced precordial leads, lateral myocardial infarction [65], RBBB, type A WPW syndrome, HCM, Duchene's muscular dystrophy, and dextrocardia among other. Although the term "hemiblock" has been very appealing, it is also inappropriate. If this original bifascicular conception of the left His system were correct, as conceived by Rosenbaum and his school, how would it be possible to explain the following facts?

**Figure 6:**
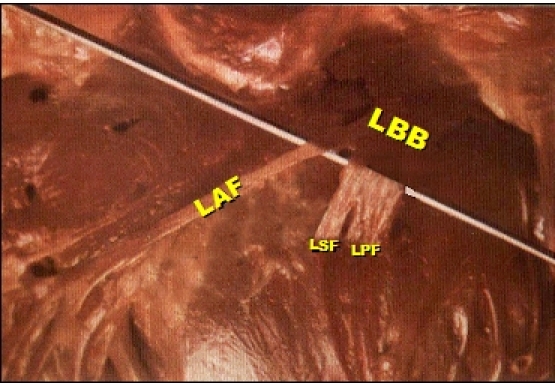
Demonstration of the type III LSF anatomic variation. Figure extracted from the original book by Rosenbaum MB, et al.  (Modified from reference number 66 with permission). The LSF clearly originates from the LPF.

- The activation of the middle third of the septum occurring 5 ms before the postero-inferior and antero-superior regions
- Rosenbaum himself said that the middle-septal activation occurred in most cases from "false tendons" anterior to the postero-inferior division. For him, the distal postero-inferior division was a fan-like structure, and its anterior "pseudo-tendons" would be responsible for the activating the middle-septal region. Currently, it is known that one of the anatomical variations of the septal division is precisely the one that depends on the postero-inferior division (10% of the cases or type III). The intermittent anterior displacement of the QRS loop in the HP in certain cases of critical injury of the anterior descending artery in the absence of other factors that may cause the same VCG changes?
- Finally, in some photographs shown in Rosenbaum's masterpiece, the presence of a third septal branch is evident [66]. (Figure 6)

In a recent review article, Dr. Marcelo V. Elizari [15] used the old terminology (hemiblocks). The author wrote:  ***"In fact the existence of middle septal fibers cannot be disregarded, and as such the functional and, probably clinical significance of the middle or septal fascicle cannot be totally ignored either"***.

In summary, we believe that the words of the Portuguese researcher Fernando de Padua are very eloquent: "IF HEMIBLOCKS DO EXIST, THEY ARE ONLY TWO - IF A THIRD ONE IS POSTULATED, HEMIBLOCKS DO NOT EXIST!" [38,39]
